# An immune biomarker associated with EMT serves as a predictor for prognosis and drug response in bladder cancer

**DOI:** 10.18632/aging.205927

**Published:** 2024-07-08

**Authors:** Yike Jiang, Zichuan Yu, Hao Zheng, Xuanrui Zhou, Minqin Zhou, Xitong Geng, Yanting Zhu, Shuhan Huang, Yiyang Gong, Liangyun Guo

**Affiliations:** 1 Department of Ultrasonography, Second Affiliated Hospital of Nanchang University, Nanchang, Jiangxi 330000, China; 2 Second College of Clinical Medicine, Nanchang University, Nanchang, Jiangxi 330000, China

**Keywords:** bladder cancer, WDHD1, immune cell infiltration, EMT, single-cell sequencing, biomarker

## Abstract

Background: Bladder cancer (BLCA), which develops from the upper endometrial of the bladder, is the sixth most prevalent cancer across the globe. WDHD1 (WD repeat and HMG-box DNA binding protein 1 gene) directly affects signaling, the cell cycle, and the development of the cell skeleton. Uncertainty surrounds WDHD1's function in BLCA immunity and prognosis, though.

Materials and Methods: Using weighed gene co-expression network analysis (WGCNA), initially, we first identified 32 risk factors in genes with differential expression for this investigation. Then, using a variety of bioinformatic techniques and experimental validation, we examined the connections between WDHD1 and BLCA expression, clinical pathological traits, WDHD1-related proteins, upper-skin-intermediate conversion (EMT), immune cell immersion, convergence factors, immune markers, and drug sensitivity.

Result: The findings demonstrated that we constructed a 32-gene risk-predicting model where WDHD1 was elevated as a representative gene expression in BLCA and related to a range of clinical traits. Furthermore, high WDHD1 expression was a standalone predictor associated with a worse survival rate. The most commonly recruited cells and their evolutionary patterns were highlighted to better comprehend WDHD1's function in cancer. High WDHD1 expression was associated with many aspects of immunology. Finally, the study found that individuals with high expression of WDHD1 were drug-sensitive to four different broad-spectrum anti-cancer drugs.

Conclusion: These results describe dynamic changes in the tumor microenvironment in BLCA and provide evidence for the hypothesis that WDHD1 is a novel biomarker of tumor development. WDHD1 may therefore be a useful target for the detection and management of BLCA.

## INTRODUCTION

Bladder cancer (BLCA) stands as the most prevalent malignancy of the urinary tract and ranks among the world’s top ten cancers. It is categorized into two primary types: muscle-invasive bladder cancers (MIBC) and non-muscle-invasive bladder cancers (NMIBC) [1]. However, even among patients getting the greatest surgery and chemotherapy, 50–70% of NMIBC patients still experience recurrence and transfer, and 10–20% continue to advance to MIBC [2, 3], resulting in a mere 23–48% overall survival percentage after 5 years. Owing to the aggressiveness and unpredictability of the disease, clinical results for BLCA patients have not much improved over the past two decades, despite improvements in surgical technology and postoperative care [4]. However, there are still few trustworthy tumor markers that can precisely forecast the emergence and development of BLCA. Therefore, it is crucial to find and research novel prognostic markers.

The tumor microenvironment (TME), which consists of immune and stem cells from non-tumor cells, is crucial in the growth and spread of diseases [5, 6], such as bladder, lung, and breast cancer [7]. Certain epithelial cells within the urinary tract possess regenerative capabilities and undergo epithelial-mesenchymal transition (EMT), a critical biological process enabling malignant tumor cells to migrate and invade [8, 9], avoiding the surveillance of the epidemic [10]. As first-line treatments for a variety of malignancies, including BLCA, immune checkpoint inhibitors (ICIs) targeting the cytotoxic T lymphocyte-associated protein 4 (CTLA-4) and programmed cell death-ligand-1 (PD-L1) pathways have been discovered. Therefore, additional research should be done on the effects of immunotherapy as well as changes in EMT in BLCA.

WDHD1 is a protein-coding gene with 1127 amino acids and a 125 kDa molecular weight. Stem cells are where it is largely expressed. WDHD1 (WD repeat and HMG-box DNA-binding protein 1) has 1127 amino acids and a molecular weight of 125 kDa. It is primarily expressed in stem cells. Studies have revealed that overexpression of WDHD1, which is crucial for chromosome formation, transcription, DNA replication, and cell death [11], has been linked to the proliferation of cancerous cells in several cancers, including non-small cell lung cancer (NSCLC) [12], esophageal cancer [11], gallbladder cancer [13], etc. Evidence suggests that WDHD1’s regulation of the cell cycle contributes to the development of laryngeal squamous cell cancer (LSCC) [13, 14]. It is unclear how WDHD1 contributes to BLCA, though.

In this work, potential genes that might be implicated in the progression of BLCA were found using WGCNA together with information from the TCGA and GEO databases. Then, we developed a risk assessment model to predict patients’ responses to immunotherapy and drug sensitivity. Screening identified WDHD1 as a representative gene; its predictive value was assessed, elucidating its primary biological function in cell proliferation and visualizing WDHD1’s dynamic evolution in EMT and its role in immune evasion. In conclusion, our research has discovered a new gene marker linked to EMT and immunity that can predict a patient’s prognosis for bladder cancer and whether they will respond to immunotherapy.

## MATERIALS AND METHODS

### Data acquisition and processing

Clinical data and RNA-seq results of BLCA patients are available in the widely used public database TCGA (https://www.cancer.gov/tcga) [15]. We acquired a single normal sample from the genotyping database derived from tumor tissues, as the TCGA database only has 701 records. Genotype-Tissue Expression (GTEx) dataset, available at https://www.gtexportal.org [16]. Then, FPKM (million fragments per million) was applied to RNA-seq.kit, and FPKM data were used to assess WDHD 1 expression in log2 style.

### Weighted gene co-expression network analysis (WGCNA)

WGCNA was utilized to determine gene clusters with a high degree of functional correlation, and we used it to figure out the optimal soft threshold for a network to be considered scale-free [17]. By analyzing connections between genes and clinical traits, genes that are highly correlated with clinical features in bladder cancer were identified. The gene connection was calculated as the Pearson correlation’s absolute value. It was discovered that the module’s hub genes were highly connected.

### Construction of a bladder cancer-related prognostic signature

LASSO-Cox regression analysis, which utilizes the least absolute shrinkage and selection operator, was performed on the prognostic candidates [18]. Furthermore, we selected the ideal penalty parameter λ, which is connected with the smallest 10-fold. Using cross-validation, we created a 32-gene optimum prognostic model. Patients with bladder cancer were divided using the median risk score to create high- and low-risk categories. To compare the overall survival (OS) of patients in the high- and low-risk groups, Kaplan-Meyer analysis (K-M) was conducted. ROC traces were displayed in the training and validation sets to judge the risk scoring model’s precision.

### LinkedOmics

Widely recognized as a comprehensive website, LinkedOmics (https://www.linkedomics.org/) is regularly utilized for the analysis of multidimensional data across 32 different types of cancer [19]. The findings from the analysis enabled us to investigate the co-expressed genes associated with WDHD1 in the TCGA BLCA database. Heat maps and volcano plots provided compelling evidence in support of this.

### GEPIA

The Gene Expression Profiling Interactive Analysis database (http://gepia.cancer-pku.cn/) was used for visualization [20]. Clinical and pathological features include the progression-free interval (PFI), overall survival (OS), and disease-specific survival (DSS).

### GSEA

GSEA was used to examine the consistency and variability of phenotypic features in order to pinpoint common or exclusive biological functions [21]. To uncover WDHD1-related BLCA pathways, we employed the GSEA tool (version 4.0.3: https://www.gsea-msigdb.org/gsea/downloads.jsp). Differences between high and low WDHD1 groups were examined to elucidate essential functional and pathway alterations. We ran GSEA on 1,000 genomic alignment analyses to obtain normalized enrichment scores (NES) using the following critical thresholds:a nominal *P*-value of 0.05 and a 0.05 FDR *q*-value.

### Single-cell sequencing analysis

Single-cell sequencing analysis was done by the R package “Seurat.” We first calculated the percentage of mitochondrial genes and set the parameters to minGene = 500, maxGene = 4000, and pctMT = 15. Next, we selected the first nine principal components for t-distributed stochastic neighborhood embedding (t-SNE) analysis [22]. Cell type annotation was performed with the R package “SingleR,” while cell track and pseudotemporal analyses were carried out using the R package “monocle.” Differential expression of genes between cell populations, types, and states was identified using the “Wilcox” method in the R package “Seurat.”

### TIMER

The Tumor Immune Estimation Resource (TIMER) is a synthetic web used for analyzing the levels of immune invasion in diverse cancers, which include 32 cancer types [23]. In this work, we use the “Diff-Exp module” to confirm the expression of WDHD1 in diverse tumors. Then the “gene module” was used to estimate the relationship between WDHD1 and immune infiltration. Finally, with the help of the “correlation module,” we verified the relationship between WDHD1 and tumor-infiltrating immune cell markers in BLCA.

### TISIDB

TISIDB (http://cis.hku.hk/TISIDB/) is a compositive online website probing the interaction between tumors and the immune system [24]. We employed it to analyze the relationship between WDHD1 and chemokines as well as immune markers in BLCA.

### GSCA analysis

GSCA (http://bioinfo.life.hust.edu.cn/GSCA) is an integrated platform for comprehensive drug and immunogenomic analysis [25]. By combining the clinical information of patients with small-molecule drugs, users can explore the relationship between gene expression and the drug sensitivity of patients for better experimental design and further clinical trials. We used it to reveal the drug sensitivity of WDHD1 to PD-0325901, RDEA119, trametinib, selumetinib, and other drugs.

### CGP2016

CGP2016 is a database and tool developed by the Cancer Genome Project at the University of Cambridge in the United Kingdom to provide a comprehensive resource for cancer genome data. The database contains a large amount of genomic data from cancer samples, including information about genetic mutations, gene expression, and chromosome variations.

### CTD

The Comparative Toxicogenomics Database (CTD, http://ctdbase.org/) is an online drug data resource that helps to study the molecular mechanisms and linkages of the effects of chemical drugs and small molecules [26]. We used it to query WDHD1 for interacting drugs or small molecules.

### Cell culture

Cell lines were purchased from the Chinese Academy of Sciences (Shanghai, China). RPMI-1640 medium was used to culture some cells, while Dulbecco’s modified Eagle’s medium (DMEM) supplemented with 10% fetal bovine serum in high glucose was used to culture other cell lines. The cells were cultured at 37°C in a humidified cell culture incubator under 5% CO_2_.

### Real-time PCR (qRT-PCR)

Total RNA was extracted according to the instructions for Trizolrogen (USA). Total RNA was then reverse transcribed to complementary DNA (cDNA) using the RT-PCR kit. Finally, the mRNA levels of the relevant genes were measured using the Real-Time qPCR kit.

### Western blotting

The RIPA protein extraction method (Beyotime, Shanghai, China) was used to extract the total protein from glioblastoma cells. Using the BCA protein assay kit (Thermo Fisher Scientific, Waltham, MA, USA), the protein content was assessed after centrifugation. The proteins were then separated using SDS-polyacrylamide gel electrophoresis (SDS-PAGE) and transferred using a PVDF membrane. After incubating with the designated antibodies overnight at 4°C, the membrane was washed three times with TBST. Subsequently, the membrane was coated with secondary antibodies conjugated to WDHD1 and incubated for an additional hour at room temperature. Finally, the AmershamTM ImageQuant 800 system (GE Healthcare, USA) was used to detect protein expression.

### Transwell assay

Cell invasion was evaluated using the Transwell chamber (Shanghai, China). Cells were seeded into the upper chamber of a 24-well Transwell chamber precoated with 1 mg/mL matrix gel in serum-free DMEM medium. When the cells reached approximately 80% confluence, DMEM medium supplemented with 10% fetal bovine serum (FBS) was added to the lower chamber, and the cells were incubated for 24 hours. Subsequently, the invaded cells were fixed with 4% paraformaldehyde and stained with 0.5% crystal violet for 30 minutes. To assess the cell invasion ability, the number of invading cells was then counted microscopically.

### Statistical analysis

R (version 4.1.2) was used for subsequent analysis. WDHD1 expression data was extracted from normal and tumor tissues of BLCA, including TCGA and GTEx. We used “ggplot2,” “patchwork,” “tidyverse,” “monocle,” “clusterprofiler,” “igraph,” and “ggsci” software packages for calculation and visualization. The Cox proportional hazards regression model was constructed using the R package “survival” to investigate the correlation between WDHD1 expression and prognosis in BLCA. Logrank tests were used for statistical analysis to obtain prognostic significance.

### Data availability statement

Publicly available datasets were analyzed in this study. The data are accessible in the TCGA and GEO databases. Further inquiries can be directed to the corresponding author.

## RESULTS

### WGCNA and identification of significant modules

**Figure 1:**
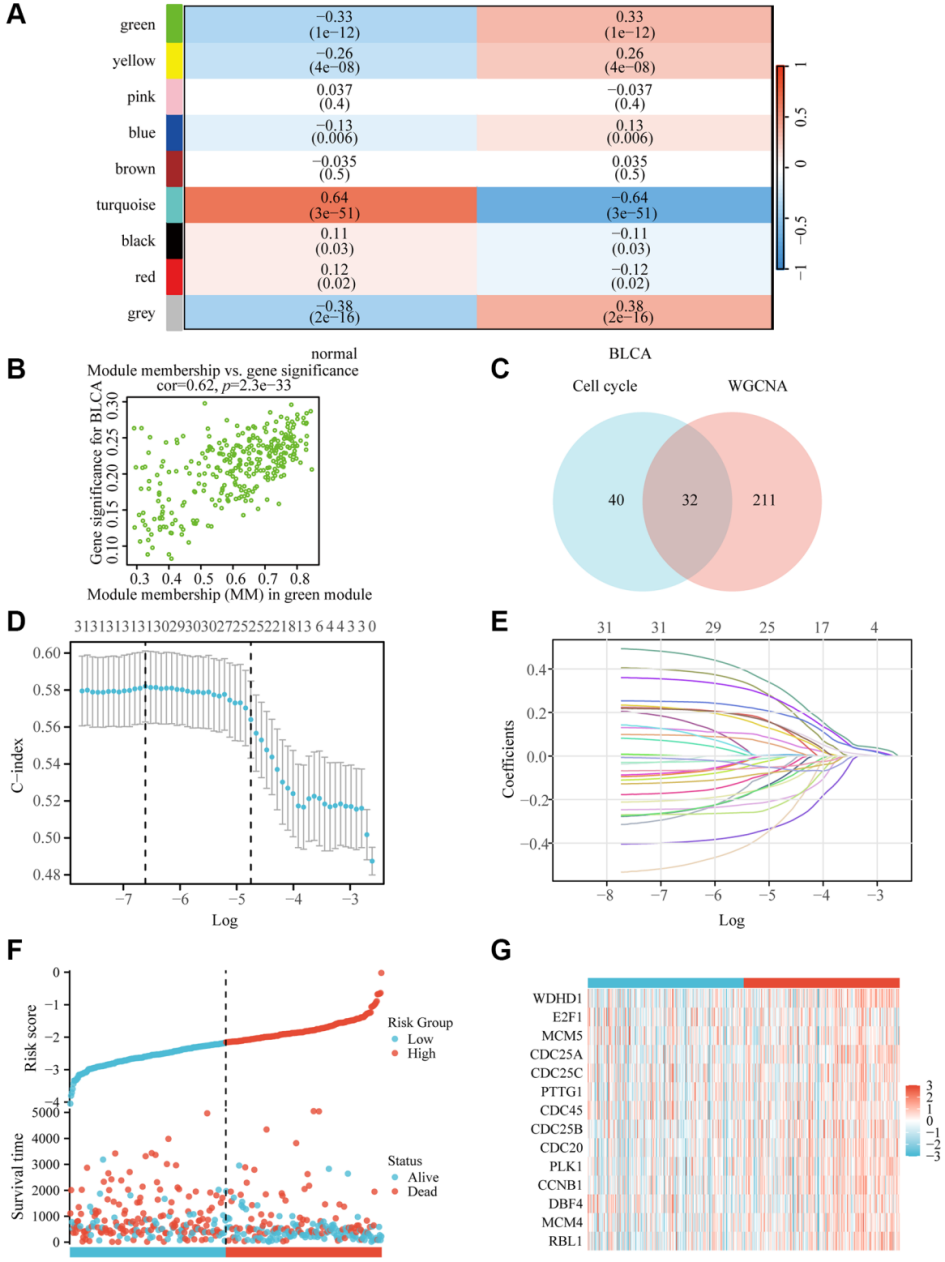
**WDHD1 gene screening and prognostic model establishment.** (**A**) Module–trait relationship heatmap. (**B**) Correlation between module members in the green module and gene significance in the BLCA. (**C**) Genes screened by WGCNA and genes associated with the cell cycle take intersections. (**D**, **E**) lambda and cvfit curves showing least absolute shrinkage and selection operator (LASSO) regression with minimum criteria. (**F**, **G**) distribution of risk scores in TCGA patients.

In order to explore the predicted risk factors in BLCA, we initially identified genes that differed in expression in bladder cancer. We set up the value of 6 as a flexible threshold for limitless network screening. The green module has the largest correlation with bladder urothelial carcinoma according to the heat map (Figure 1A), and according to the genetic distribution of the green modulus, bladder cancer is strongly correlated with the members of the module (Figure 1B). Finally, we were able to locate 32 nodes of the gene using the Venn diagram (Figure 1C).

### Construction of BLCA-related genes and analysis of independent prognostic potential.

**Figure 2:**
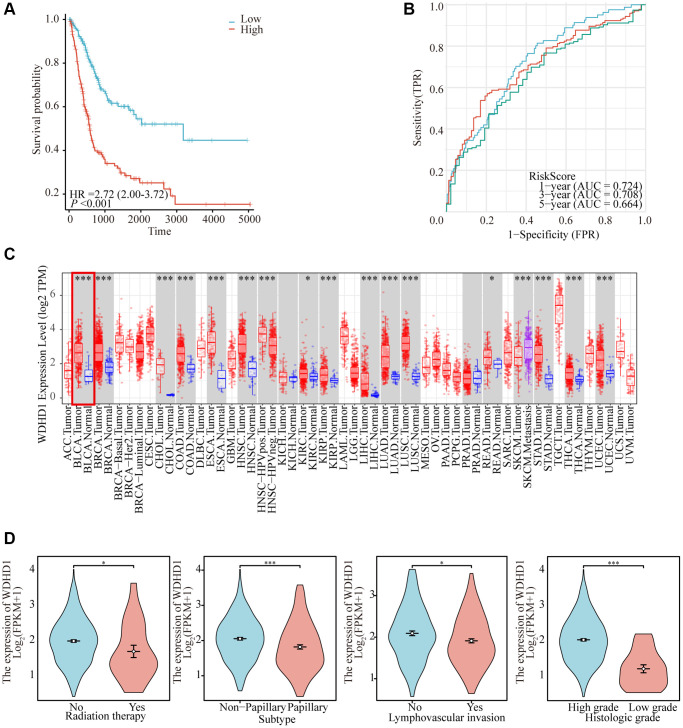
**Comprehensive analysis of model reliability and gene expression.** (**A**) Kaplan-Meier curves for overall survival in the TCGA-BLCA cohort for the low-risk and high-risk groups. (**B**) Time-dependent ROC analysis curves for 1-, 3-, and 5-year OS characteristics in the TCGA dataset. (**C**) Expression of WDHD1 in BLCA and paracancerous tissues (**D**) WDHD1 expression and clinicopathologic features.

To construct a genomic landscape related to bladder cancer (BLCA) and analyze its potential for independent prognostic significance, we delved into the cross-gene correlations between BLCA-specific genes and those implicated in the cell cycle utilizing LASSO-Cox regression. This analysis facilitated the creation of a risk model to predict overall survival, with calibration of the C-index and lambda values illustrated in Figure 1D, 1E. Based on middle-risk evaluations, they were divided into high-risk and low-risk groups (Figure 1F, 1G), with low-risk patients having considerably higher survival rates (Figure 2A). According to Figure 2B, the risk model’s AUC at 1, 3, and 5 years was 0.724, 0.708, and 0.664, showing that the risk model is capable of accurately predicting the prognosis of BLCA patients.

### WDHD1 expression and predictive meaning in BLCA

**Figure 3:**
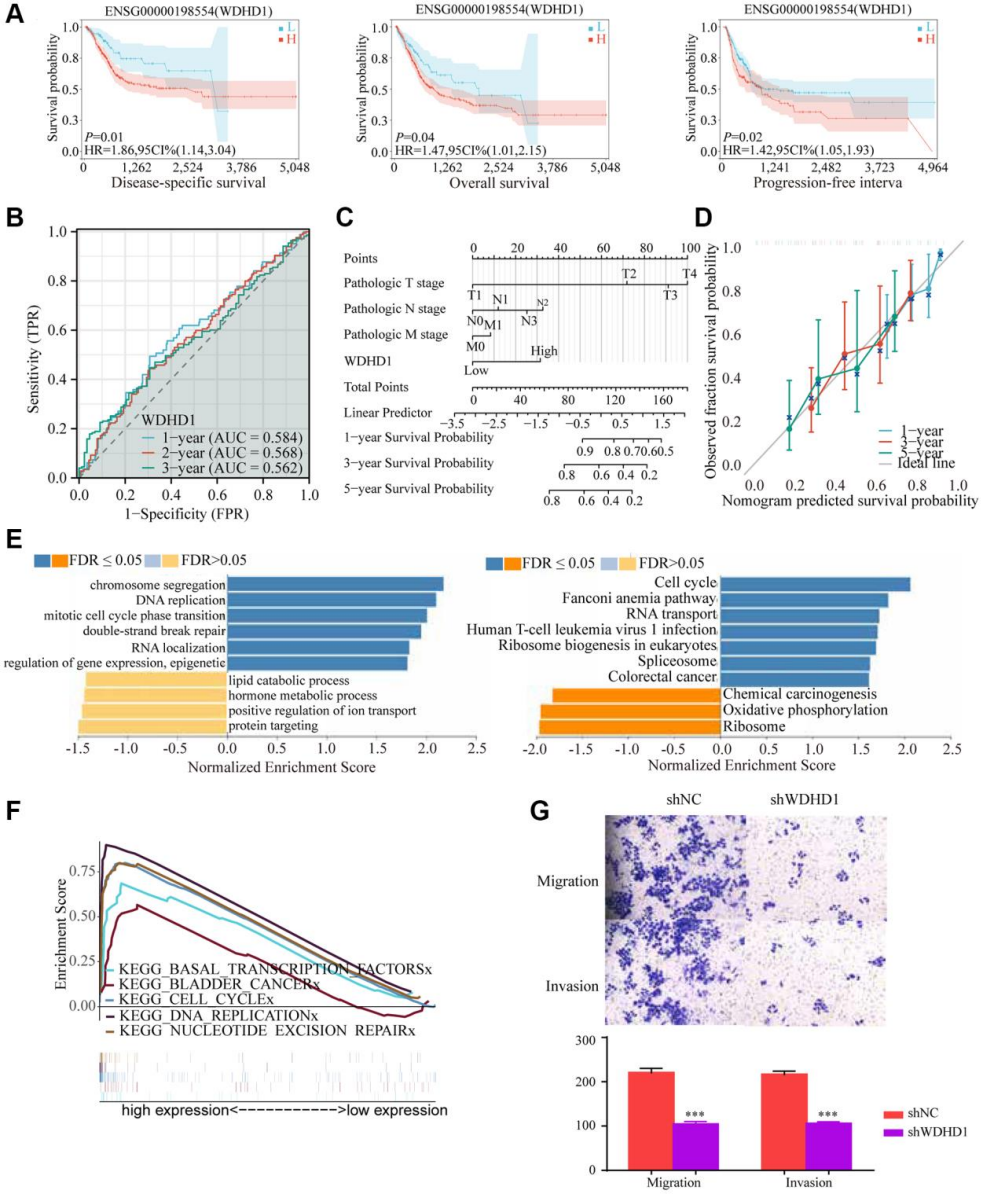
**Prognostic value and functional analysis of WDHD1 in BLCA.** (**A**) Prognostic modeling assays. (**B**) Time-dependent ROC analysis curves for 1-, 3-, and 5-year OS characteristics of WDHD1 (**C**, **D**) Nomogram and ROC curves for predicting the 1-, 3-, and 5-year OS of BLCA patients in the entire cohort. (**E**) GO and KEGG of WDHD1. (**F**) GSEA analysis of the correlation between DEGs of WDHD1 and function pathways. (**G**) Invasion and migration assay, knockdown of WDHD1 expression reduces invasion and migration ability.

In order to investigate the differential expression of WDHD1, we examined the expression in both cancer and cancer-side tissues (Figure 2C). Additionally, we explored WDHD1’s expression alterations in BLCA samples with different clinical and pathological features (Figure 2D). The results of WDHD1’s expression on prognosis showed that WDHD1’s high expression was associated with poor prognosis (Figure 3A), and the model’s accuracy was evaluated using the ROC curve. (Figure 3B). Then, using nomogram and predictive calibrated curve as described in the BLCA (Figure 3C, 3D), where WDHD1 is an independent prognosis factor, we created a nomogram to further analyze forecast survival rates in patients with various clinical pathological characteristics and low WDHD1 expression.

### Functional significance of WDHD1

We examined the correlation gene of WDHD1 for a deeper comprehension of the biological importance of WDHD1 in BLCA. GO and KEGG function-rich analysis demonstrated that the correlation genes of WDHD1 existed in the cell cycle and DNA replication. Gene set enrichment analysis (GSEA) found the DEGs were enriched in bladder cancer, cell cycle, and DNA replication (Figure 3E, 3F). Finally, we performed an invasive migration experiment to investigate the effect of WDHD1 expression on bladder cancer cells for invasiveness and migration. The results revealed that high WDHD1 expression was supportive of increased bladder cancer cells’ capacity for invasive migration (Figure 3G).

### WDHD1 positioning and immersion analysis in a tumor microenvironment: combining single-cell sequencing analysis and estimate algorithms

**Figure 4:**
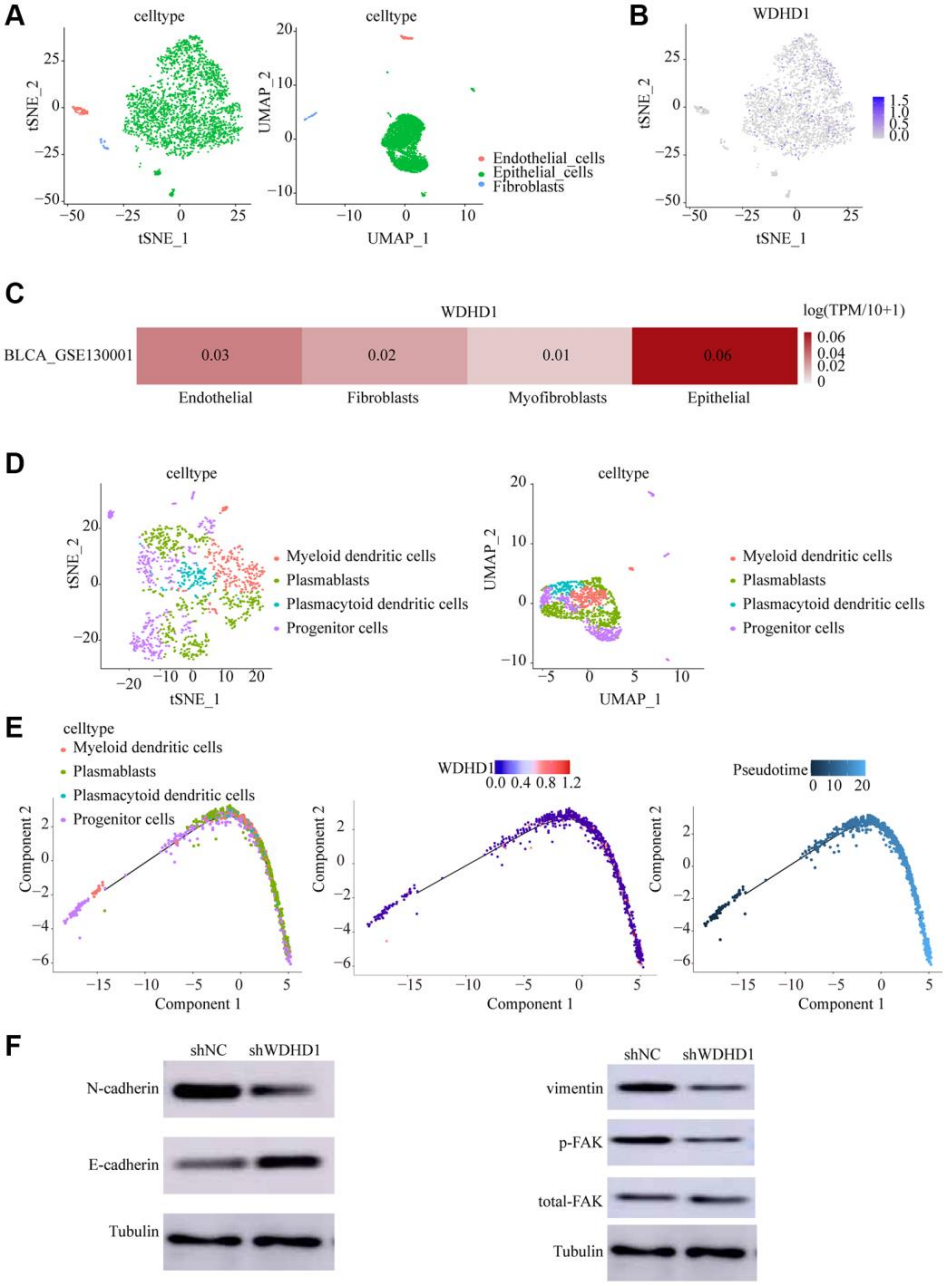
**Cell dynamics in the progression of bladder cancer.** (**A**, **B**) tSNE and UMAP cell clustering analysis to distinguish cell types in the tumor microenvironment. (**C**, **D**) Tumor cells showing WDHD1 expression sites. (**D**) epithelial cells were re-clustered using tSNE and UMAP. (**E**) Changes of cell types and WDHD1 expression with pseudotime analysis. (**F**) Western blot showed that decreased expression of WDHD1 inhibited the expression of N-Cadherin,Vimentin, phosphorylated focal adhesion kinase (p-FAK) .The tubulin was used as loading control. ^*^p < 0.05, ^**^p < 0.01

In order to study changes in the tumor microenvironment during the upper-intermediate cell conversion process, we discovered that WDHD1 is expressed in a variety of primary cells (Figure 4C). Upper-intermediate cell transformation is a crucial biological process in the development of scalp cancer. Identifying cells by tSNE and UMAP, we discovered that WDHD1 was largely expressed in malignant cells (Figure 4A, 4B). Furthermore, we reclassified the epithelial cell (Figure 4D). By pseudo-time trajectory analysis, we found that the tumor microenvironment matures with increasing WDHD1 expression in a wide range of cells, a process that correlates with the progenitors of a wide range of immune cells (Figure 4E). The knockdown of WDHD1 resulted in the elevation of E-calmodulin and down-regulation of N-calmodulin, vimentin, and p-FAK, and WDHD1 was not only highly expressed in epithelial cells but also regulated EMT-related processes (Figure 4F). WDHD1 may have contributed to the development of the immunological and tumor microenvironments.

### Relationship of WDHD1 with the immune microenvironment, immune immersion, and immune markers

**Figure 5:**
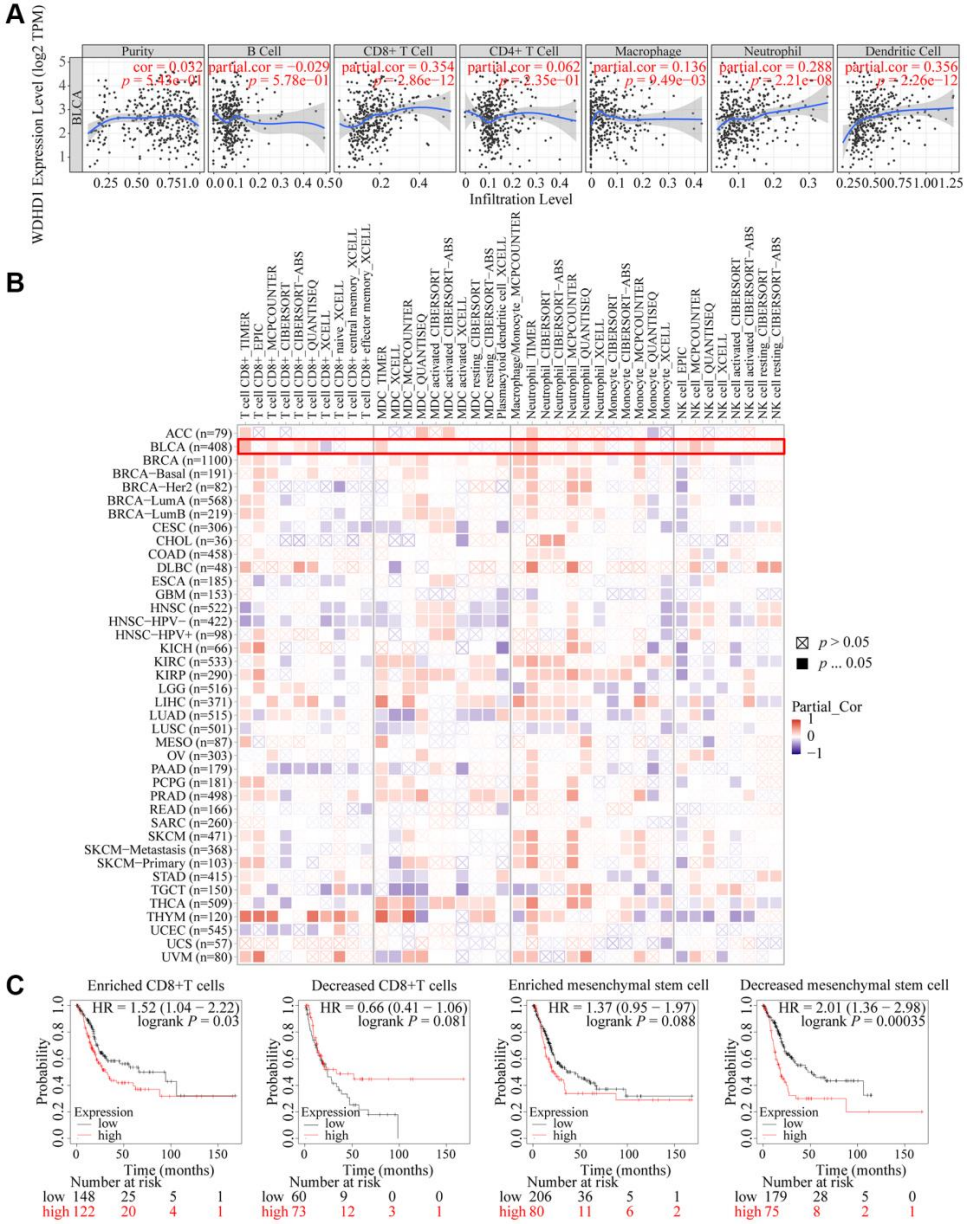
**Landscape of immune infiltration and survival in BLCA.** (**A**) Correlation of WDHD1 expression in BLCA with tumor purity and infiltration of different immune cells. (**B**) Correlation of WDHD1 expression with different subtypes and different algorithms of immune cell infiltration. (**C**) Prognosis of BLCA patients with WDHD1 expression in high/low infiltration of CD8+ T cells and MSCs.

**Figure 6:**
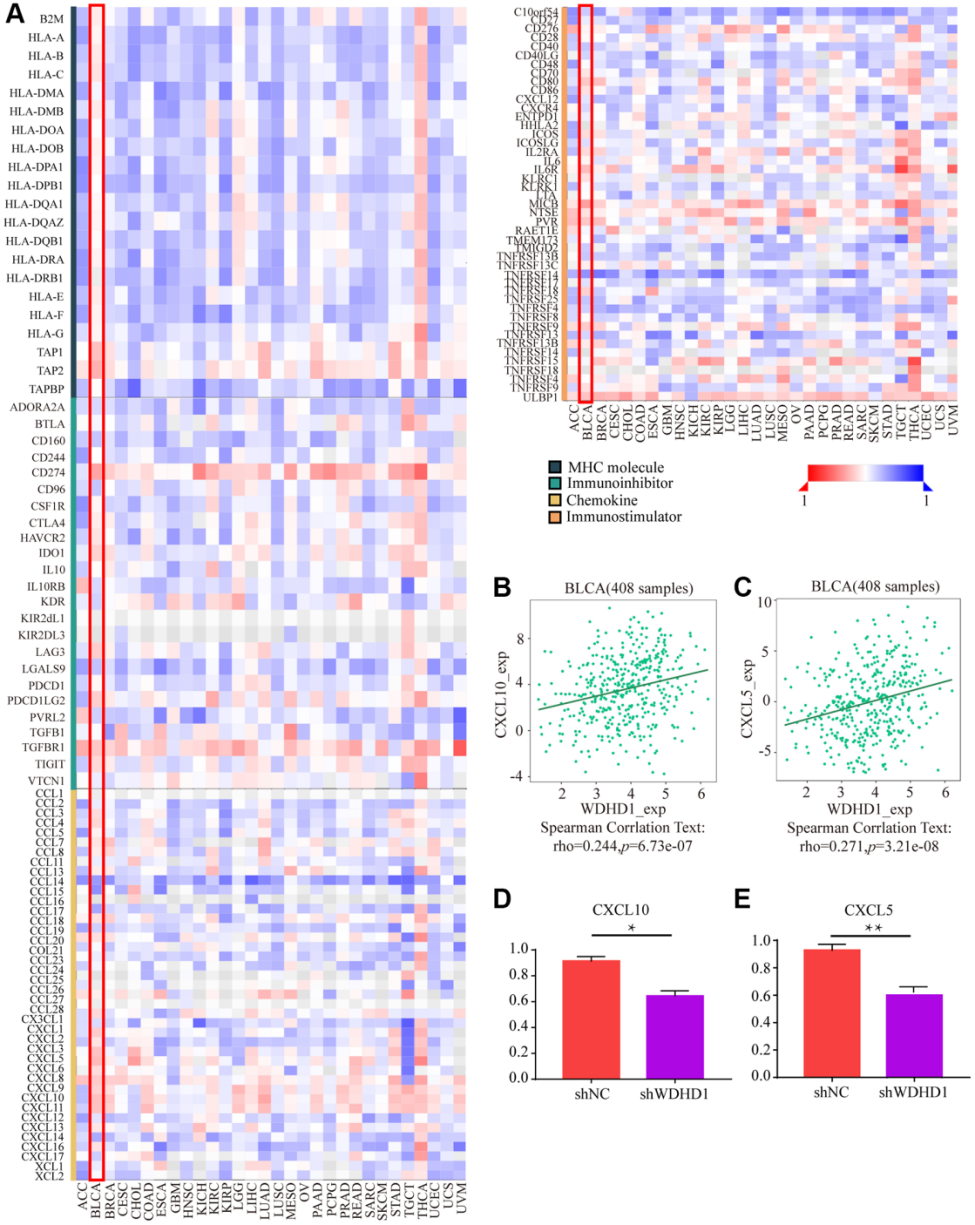
**The effect of WDHD1 on immunological status in pan-cancers using the TISIDB.** (**A**) Correlation between WDHD1 and immunomodulators (chemokines, MHC, immune stimulators and receptors). The color indicates the correlation coefficient. (**B**–**E**) Correlation of WDHD1 with the expression of chemokines CXCL10 and CXCL5.

The tumor microenvironment (TME) is essential for the development, growth, and dissemination of malignancies. We defined the characteristics of immersion in various immune cells. The results showed that WDHD1 was linked to the absence of immune cells invading the tumor, including CD8+ T cells, neutrophils, macrophages, and dendritic cells (Figure 5A, 5B). In the CD8+ T cell enrichment model, patients with high WDHD1 expression had a poor prognosis, while in patients not enriched with CD8+ T cells, WDHD1 expression had no significant effect on the survival of BLCA patients. Meanwhile, the prognosis of BLCA patients enriched with mesenchymal stem cells does not depend on the expression of WDHD1, and BLCA patients who are not enriched with mesenchymal stem cells will have a poor prognosis due to high expression of WDHD1(Figure 5C). Additionally, WDHD1’s CNV was connected to various immune cell invasions (Supplementary Figure 1A). Meanwhile, we analyzed the mRNA (protein) expression patterns of WDHD1 in different immune subtypes, and the outcomes revealed significant differences (Supplementary Figure 1B). As shown in Supplementary Figure 1C–1F, the survival model of BLCA patients with high expression of WDHD1 while enriching Th1 cells, B cells, CD4+ memory T cells or regulatory T cells can significantly reduce the survival of BLCA patients. By analyzing the correlation between WDHD1 and four categories of immunomodulators, we revealed that WDHD1 was associated with MHC, immunostimulants, immunosuppressants, and convergence factors in the majority of cancers. Immune checkpoints are known to be involved in tumor immune escape. Additionally, WDHD1 is strongly related to BLCA-related convergence factors: CXCL10 and CXCL5 (Figure 6A–6C). The expression of the convergence factor was drastically reduced in shWDHD1 cells (Figure 6D, 6E). Overall, WDHD1 may have an impact on the expression of convergence factors, immunological immersion, and immune survival.

### Predictive and therapeutic drug sensitivity analysis of WDHD1-related immune responses in immunotherapy

**Figure 7:**
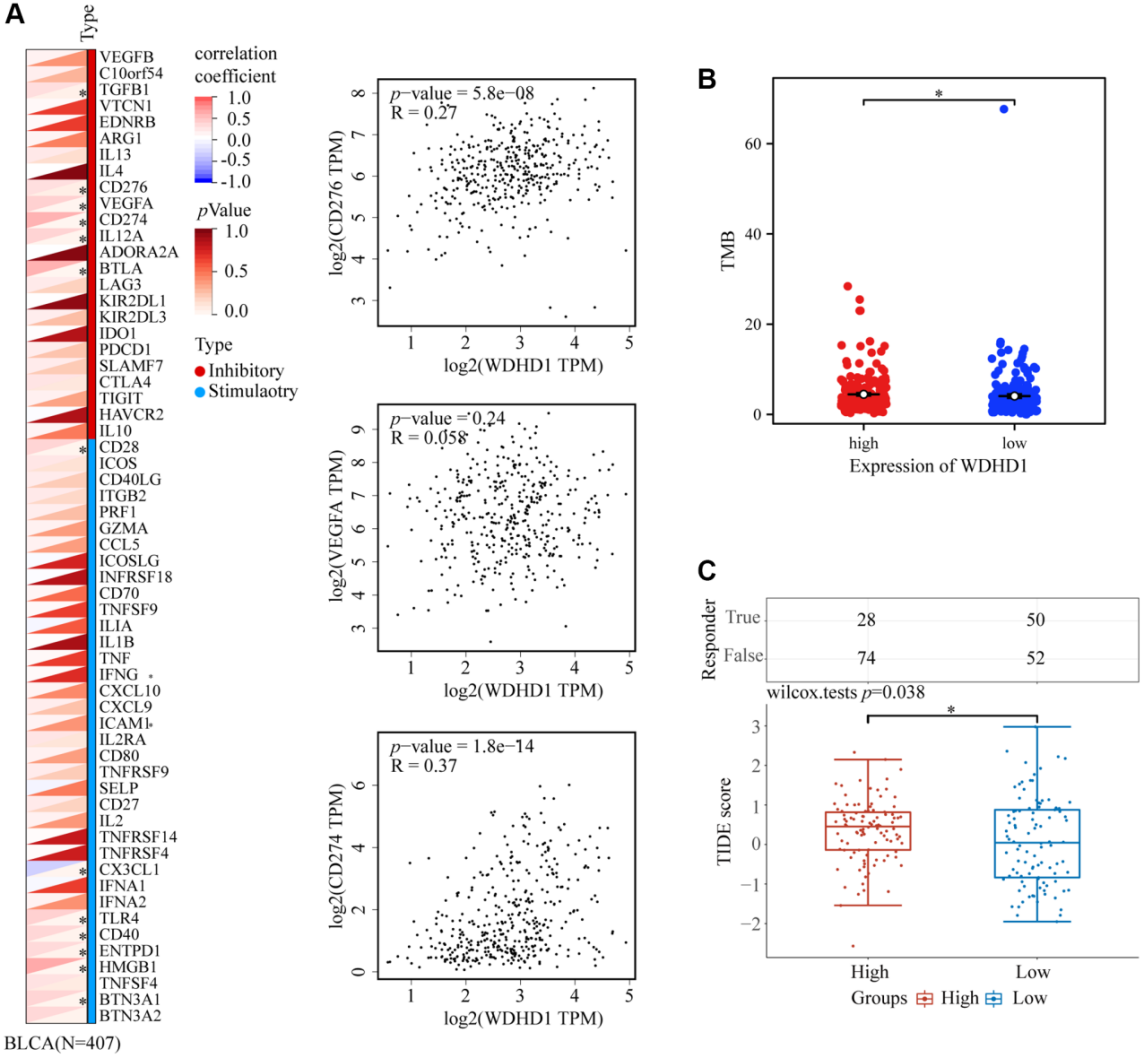
**WDHD1-related immune responses in immunotherapy.** (**A**) Heatmap of the correlation between WDHD1 and immune checkpoints. (**B**) Heatmap of correlation between WDHD1 and TMB from TCGA database. (**C**) Histograms of WDHD1-associated ICB treatment efficacy between WDHD1 high-expression and low-expression groups were obtained from the TCGA database by the TIDE algorithm. Low scores indicate good efficacy.

**Figure 8:**
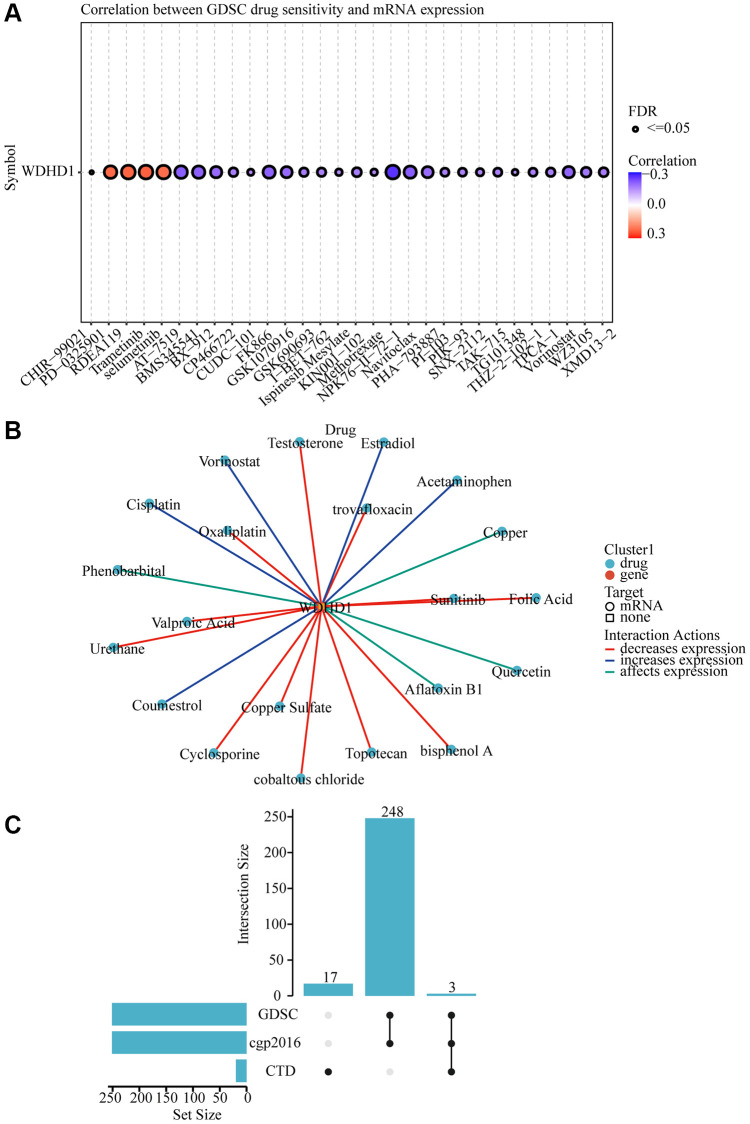
**WDHD1-related drug molecular effects.** (**A**) Drug sensitivity analysis of various drugs. (**B**) Visualization of drug-gene interactions. (**C**) WDHD1-upset.

To explore the antigenicity of tumor cells changed with WDHD1. We discovered that greater levels of TMB were associated with its expression in BLCA (Figure 7B). Particularly, CD276, VEGFA, and CD274 all exhibited a high connection with WDHD1 (Figure 7A). We calculated a TIDE score, which was higher for the WDHD1 high-expression group in the BLCA (Figure 7C). Additionally, patients with high expression of WDHD1 were sensitive to four broad-spectrum anti-cancer medicines: PD-0325901, RDEA119, trametinib, and selumetinib (Figure 8A). Furthermore, we found that there were 21 drugs with effects on the expression of WDHD1, and 12 of them showed inhibition effect (Figure 8B). Using the drug intersection of GDSC, cgp2016 and CTD, we found a series of drugs that may be helpful for the clinical application of BLCA (Figure 8C). In conclusion, research on WDHD1 may support immunotherapy for BLCA and offer advice to clinicians regarding medication.

## DISCUSSION

Bladder cancer is one of the most common malignant tumors of the urinary system and is highly heterogeneous. Patients with BLCA only have a 23% to 48% 5-year survival rate. The major method of treating bladder cancer in recent years has been surgery combined with chemotherapy and immunotherapy, although BLCA recurrence and transmission rates are still very high. Depending on the stage of EMT, tumor cells perform several different roles in the development and resistance to therapy of metastases [27]. The relationship between tumor stem cells, EMTs, and TMEs is becoming increasingly obvious, and it is unknown how these roles in bladder cancer are associated with biomarkers [28]. The cell morphology changed and showed a shuttle shape with an increase in N-calmodulin [29, 30] and a decrease in E-calmodulin. The epithelial cells acquired the characteristics of mesenchymal cells, while cell motility and invasive migration ability were significantly improved. Stromal cells play a significant part in TME [31]. For instance, tumor-related fibrous cells may release HGF, a tumor mortality factor, to encourage the growth of the tumor microenvironment [32, 33], while CD8+ T lymphocytes may secrete cell factors to regulate tumor progression, create blood vessels, and build the tumor microenvironment [34–36].

WD Repeat and HMG-Box DNA Binding Protein 1 (WDHD1) can act as an essential module for signaling and cellular skeletal regulation, which has multiple N-terminal WD40 domains and C-terminal high mobility group (HMG) boxes. Multiple nucleoproteins involved in chromosomal assembly and replication utilize the HMG box. Numerous studies have demonstrated that WDHD1 can affect the growth of esophageal cancer by regulating the PI3K/AKT pathway during the cell cycle, WDHD1 in nasopharyngeal cancer, and ITGAV in the management of the NPC cell cycle. Research has also indicated that microRNA-494 may impede upper-intercutancy transformation by WDHD1 expression in gallbladder cancer by lowering its level of expression [13]. It is unclear how WDHD1 is expressed and functions in BLCA, though.

Using cell cycle-related genes and WGCNA data, we first identified the risk-predicting genes for bladder cancer in our study. Then, we created a risk prediction model by screening the 32 genes that were chosen, demonstrating that the prognosis of the patient may be precisely predicted by our prediction model. Following the selection of the representative gene WDHD1 in the predictive model, clinical pathological characteristics, and prognosis analysis, we established WDHD1 as an independent predictor for bladder malignancies. This was done by doing univariate and multivariate analyses. In vitro research has demonstrated that the expression of WDHD1 can have a negative regulatory influence on the proliferation and invasive migratory abilities of bladder malignancies. KEGG and GSEA results revealed that WDHD1 was enriched in the cell cycle, bladder cancer, P53, and other pathways. Pseudotime analysis with Monocle algorithms can show the dynamics of single-cell gene expression from a number of cellular functional activities, including cell differentiation, proliferation, and malignant transformation [37]. We identified WDHD1 expression mostly in epithelial cells using single-cell sequencing techniques, and then, in order to investigate the role of WDHD1 in modulating expression during the process of stem cell generation in bladder cancer, we first employed the pseudotime axis to filter it for expression. We discovered that WDHD1 expression gradually increased as the cells transformed, and the major cell types were also altered. In trials using E-calcium, N-calcium glucose, vimentin, and p-FAK, WDHD1’s capacity to take part in EMT regulation was validated. WDHD1 is assumed to be involved in the process of EMT.

We discovered that WDHD1’s expression and mutation can control the degree of immersion in various immune cells and their subtypes, CD8+ T cells, further investigating the specific TME composition and, based on its investigation, new targets that block treatment at the immune checkpoint. Existing research has demonstrated that the prognosis of bladder cancer patients is influenced by the immersion condition of CD8+ T cells, neutrophils, and macrophages. It is well established that various cells within CD8+ T cells [38, 39], as well as the TME, can impact a patient’s prognosis. We hypothesize that one of the mechanisms by which WDHD1 affects BLCA survival is by controlling the immune cell immersion because the immune survival results showed that the immersion of CD8+T cells can affect the patient’s survival. Additionally, because WDHD1 expression can affect the patient’s prognosis on its own, we believe that this hypothesis is supported. By controlling the immune response, cell proliferation, and other processes, cellular variables also have a significant impact on the emergence of bladder cancer and EMT [40].

The development of bladder cancer can be aided by a number of cell factors that CD8+ T cells can release. The expression of cell factor, a cell factor secreted by CD8+ T lymphocytes when WDHD1 is high, was therefore investigated. By secreting chemicals like IFN-γ, CD8+T cells in bladder cancer are able to directly block tumor growth [41]. Our results demonstrated a correlation between high levels of WDHD1 expression and positive CXCL10 and CXCL5 in gliomas. Therefore, we hypothesize that elevated WDHD1 expression may impact immune cells like CD8+ T cells and secrete IL-6 and TGF-β, which may result in people with bladder cancer having a dismal prognosis. In addition, we believe this gives our research into precise, tailored immunotherapy for bladder cancer a clear direction. It turns out that investigating the relationship between convergence factor and convergence factor receptor modulation in the treatment of bladder cancer is also progressively emerging as a key concept. Additionally, the primary method of current immunotherapy involves blocking immunosurveillance proteins like PD-1 and PD-L1. It is feasible to see that WDHD1 has a positive correlation with the expression of multiple immune checkpoints by studying the association between WDHD1 and the expression of different immune checkpoints in BLCA. The effects of PD-1/PD-L1 blocking therapy are closely correlated with TMB, which is frequently used to accurately predict the outcomes of immunotherapy [42, 43]. The elevated expression of WDHD1 in the immune checkpoint in cancer tissue also suggests that tumor tissue has a greater capacity for immune escape than normal cells. Currently, the effects of immune checkpoint-blocking treatments are frequently accompanied by complicated combinations of molecular processes, whereas the effects of immunotherapy are mostly influenced by individual variances in CD5 proteins in arboric cells [44]. In conclusion, our data support the idea that immunotherapy is less successful when WDHD1 expression is high and offer knowledge for investigating the molecular mechanisms and medical treatments connected to WDHD1. According to our TIDE results, the immune treatment of bladder cancer is ineffective in cases of high expression of WDHD1. The findings supported the aforementioned notion and also imply that we can successfully reduce WDHD1 overexpression in bladder cancer, which may enhance the effectiveness of immune therapy and raise the likelihood that the patient will survive. Clinical medication sensitivity in patients can be significantly impacted by genetic changes in cancer tissue, and the GDSC database offers reliable information on drug sensitivity [45]. We discovered that when WDHD1 is high, patients are responsive to the four spectral anti-cancer medications PD-0325901, RDEA119, trametinib, and selumetinib. Among them, PD-0325901 is used to treat late-stage melanoma, breast, colon, and other cancers [46]; RDEA119 is used to treat thyroid and pancreatic cancers [47–49]; trametinib is used to treat melanoma and pulmonary ovarian cancer [50]; and selumetinib has been extensively used to treat thorn neurofibroma [51, 52]. Few of the aforementioned medications, meanwhile, are used by bladder cancer patients in clinical settings. This shows that by determining the range of WDHD1 expression, we can provide patients with bladder cancer with a whole new method of clinical treatment.

## CONCLUSION

In conclusion, this is the first investigation into WDHD1 and bladder cancer. We have developed a powerful predictive model for bladder cancers using a bioinformatics method, identified a novel independent prognostic factor that is strongly related to TME and EMT of bladder cancer, and proposed a completely new strategy for immunological and pharmaceutical therapy for BLCA.

## Supplementary Materials

Supplementary Figure 1
