# Supplementary material for: An immune biomarker associated with EMT serves as a predictor for prognosis and drug response in bladder cancer
Source: Aging (Albany NY). 2024 Jul 8;16(13):10813–31. doi: 10.18632/aging.205927 (PMC11272103; doi:10.18632/aging.205927)
Supplement: Supplementary Figure 1 [file aging-16-205927-s001.pdf]

SUPPLEMENTARY FIGURE

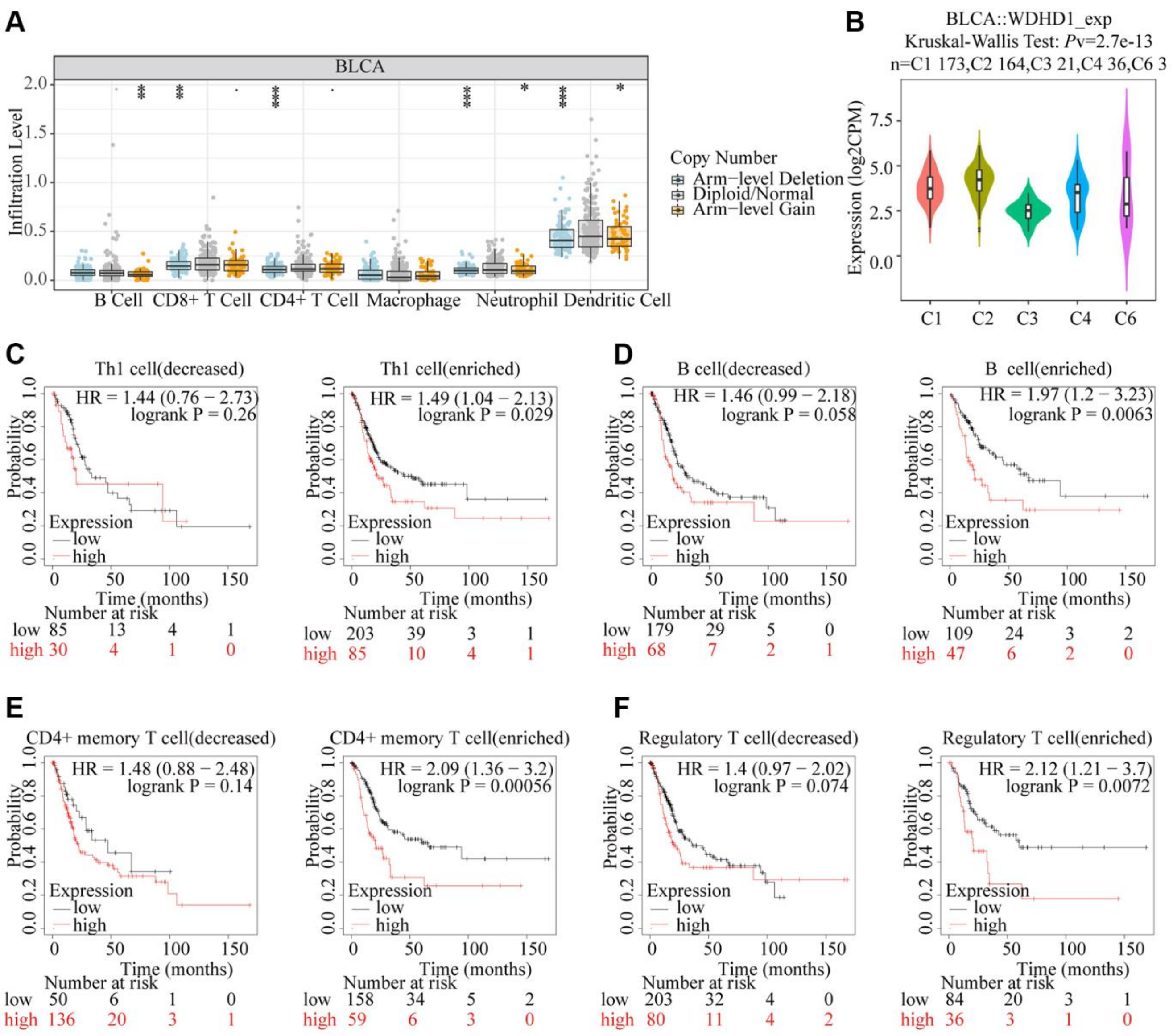

Supplementary Figure 1. Associations among WDHD1, tumor immune infiltrating cells and prognosis. (**A**) WDHD1 CNV affects the infiltrating levels of B cell, CD4+ T cell, macrophages, neutrophils, and dendritic cell in BLCA. (**B**) WDHD1 mRNA expression in different immune subtypes in BLCA. (**C**–**F**) Prognosis of BLCA patients with WDHD1 expression at high/low infiltration of Th1 cells, B cells, CD4+ memory T cells and regulatory T cells.
